# Panobinostat Synergizes with Chemotherapeutic Agents and Improves Efficacy of Standard-of-Care Chemotherapy Combinations in Ewing Sarcoma Cells

**DOI:** 10.3390/cancers16213565

**Published:** 2024-10-23

**Authors:** Kaitlyn H. Smith, Erin M. Trovillion, Chloe Sholler, Divya Gandra, Kimberly Q. McKinney, David Mulama, Karl J. Dykema, Abhinav B. Nagulapally, Javier Oesterheld, Giselle L. Saulnier Sholler

**Affiliations:** 1 Levine Cancer Institute, Atrium Health Carolinas Medical Center, Charlotte, NC 28204, USA; 2 Levine Children’s Hospital, Charlotte, NC 28203, USA; erin.trovillion@atriumhealth.org (E.M.T.);; 3 Penn State Hershey Children’s Hospital, Hershey, PA 17033, USA

**Keywords:** Ewing sarcoma, histone deacetylase inhibition, DNA damage, chemotherapy synergism, pediatric oncology

## Abstract

**Background:** The survival rate of patients with Ewing sarcoma (EWS) has seen very little improvement over the past several decades and remains dismal for those with recurrent or metastatic disease. HDAC2, ALK, JAK1, and CDK4 were identified as potential targets using RNA sequencing performed on EWS patient tumors with the bioinformatic analysis of gene expression. **Methods/Results:** The pan-HDAC inhibitor Panobinostat was cytotoxic to all the Ewing sarcoma cell lines tested. Mechanistically, Panobinostat decreases the expression of proteins involved in the cell cycle, including Cyclin D1 and phospho-Rb, and DNA damage repair, including CHK1. Further, Panobinostat induces a G1 cell cycle arrest. The combination of Panobinostat with Doxorubicin or Etoposide, both of which are used as standard of care in upfront treatment, leads to a synergistic effect in EWS cells. The combination of Panobinostat and Doxorubicin induces an accumulation of DNA damage, a decrease in the expression of DNA damage repair proteins CHK1 and CHK2, and an increase in caspase 3 cleavage. The addition of Panobinostat to standard-of-care chemotherapy combinations significantly reduces cell viability compared to that of chemotherapy alone. **Conclusions:** Overall, our data indicate that HDAC2 is overexpressed in many EWS tumor samples and HDAC inhibition is effective in targeting EWS cells, alone and in combination with standard-of-care chemotherapy agents. This work suggests that the addition of an HDAC inhibitor to upfront treatment may improve response.

## Simple Summary

There has been little improvement in Ewing sarcoma survival rates over the past several decades and new therapeutic options are desperately needed. Histone deacetylases (HDACs) are known to play a role in cancer development and progression. We show that HDAC2 is overexpressed in Ewing sarcoma cells and that an HDAC inhibitor, Panobinostat, is cytotoxic to Ewing sarcoma cells both alone and when combined with standard of care. Mechanistically, we show that Panobinostat increases the DNA damage caused by standard of care leading to the death of the Ewing sarcoma cells. We believe that HDAC inhibition combined with standard-of-care chemotherapeutics may be an effective treatment option for Ewing sarcoma patients.

## 1. Introduction

Ewing sarcoma (EWS) is the second most common malignant bone tumor in 10- to 19-year-olds, affecting approximately 225 children and adolescents per year in the United States [1]. The most frequent primary tumor locations are the lower extremity and pelvic bones, with the lungs, bone, and bone marrow being the most common metastatic sites [2]. The EWS-FLI1 fusion is identified in approximately 85% of tumors and other alterations, including the EWS-ERG fusion, account for the remaining 15% [1]. Current standard therapy involves compression interval neo-adjuvant chemotherapy using alternating cycles of Vincristine, Doxorubicin, Cyclophosphamide (VDC) with Ifosfamide, and Etoposide (IE); local control with surgical resection and/or radiation therapy; followed by compression interval adjuvant chemotherapy with VDC/IE [3]. The overall survival rates are approximately 75% for localized disease, and approximately 30% for metastatic disease [4]. After isolated lung-only relapse, the overall survival rate decreases to less than 30%, and less than 20% when relapsed disease involves the bone and bone marrow [1]. Despite advances, there have been limited improvements in the overall survival for EWS, specifically metastatic EWS, in the past 17 years, highlighting the need for novel therapeutic approaches.

The understanding of cancer genomics is shaping the approach to cancer treatment in pediatric and adult oncology patients [5,6,7,8,9,10]. With significant heterogeneity in gene alterations driving cancer development, novel therapies including immunotherapy and targeted agents are being discovered and implemented to improve patient outcomes. Genomic sequencing enables the identification of tumor-specific targetable mutations, mutational signatures within the tumor, and tumor-specific antigens [11]. This information can allow for a personalized medicine approach to patient care, potentially predicting therapeutic responses to inhibitors or identifying cancer-specific proteins to help design personalized anticancer vaccines.

As with most pediatric cancers, EWS tumors have a low mutational rate of 0.15/megabases [12]. The most identified genomic change is the EWSR1-FLI1 fusion; however, other genomic changes including STAG2 are found in approximately 15% of tumors, as well as CDKN2A and TP53 [13]. Copy number alterations are found in EWS, including gains in chromosomes 1q, 2, and 8 and deletions in 16q. Germline mutations in cancer predisposition genes, including TP53, BRCA, PTPN11, and PMS2, are found in 10% to 13% of patients with EWS [14]. Transcriptome analyses by RNA sequencing have also identified that the high expression of Neurexin-1 is associated with poor event-free and overall survival [15], and the high expression of *IGF2* is associated with a shorter overall survival [16]. In addition, the dysregulation of epigenetic signaling resulting in aberrant histone acetylation or deacetylation has been implicated in sarcomas [17].

The dysregulation of epigenetic signaling observed in cancer has led to the development of histone deacetylase (HDAC) inhibitors [18]. HDACs are known to play roles in cancer cell proliferation and apoptosis, making these proteins attractive targets for cancer therapy [19]. Multiple HDAC inhibitors are in use in varying types of cancer and their mechanism of action can be variable depending on cancer type, making investigating these drugs across multiple pathologies of value [20,21].

Although the EWSR1 fusions, most commonly EWS-FLI1, are known to drive cancer, there are currently no FDA-approved EWS-FLI1 inhibitors available, and targeting these fusions is a challenge [22]. With a better understanding of the DNA and RNA changes found in patient EWS tumors, we believe we can identify novel therapeutic targets and drug combinations that can be further tested to improve the outcome of EWS patients.

## 2. Methods

### 2.1. Cell Lines and Culture

**Figure 1:**
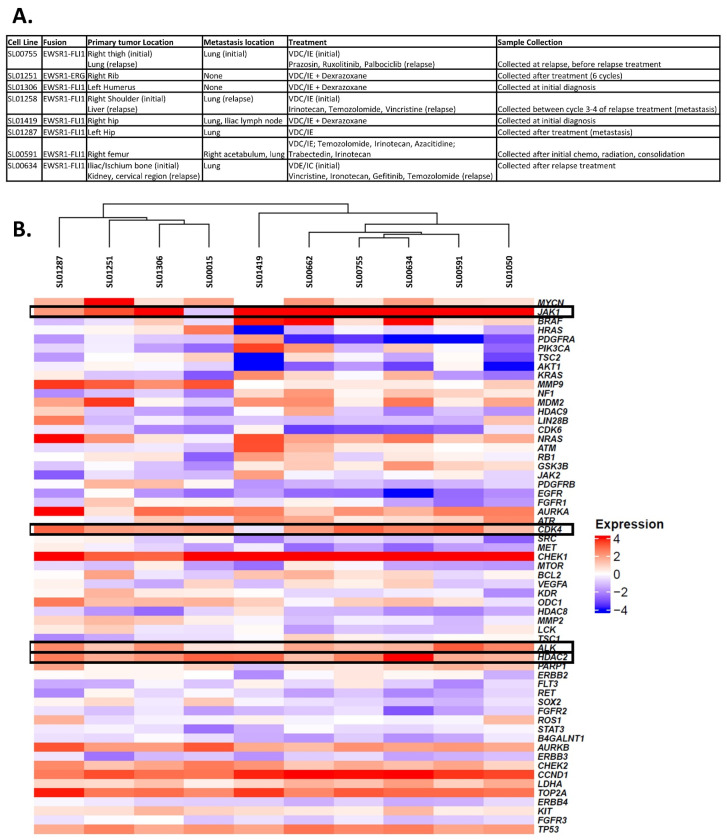
Ewing sarcoma cells overexpress HDAC2, ALK, CDK4, and JAK1. (**A**) Table of characteristics for each cell line used. (**B**) RNA expression Z-scores indicating over-/underexpressed genes.

EWS tumors from pediatric patients were obtained from Beat Childhood Cancer Research Consortium clinical trials. All the patients consented to cell line generation for use in future research. For cell line generation, tumor tissue was dissociated by mincing with a scalpel in a 10 cm dish. Complete media (MEM-α with 10% FBS, 100 U/mL Penicillin, and 100 µg/mL Streptomycin) was added to the dissociated tissue and this was placed in a 37 °C incubator with 5% CO_2_ for 25 min. After 25 min, the cells and media were transferred to a new T25 vented flask and placed in the incubator (37 °C incubator with 5% CO_2_) for growth. The cell lines generated from these tumors were maintained in MEM-α with 10% FBS, 100 U/mL Penicillin, and 100 µg/mL Streptomycin. The cell lines were authenticated by IDEXX and validated by staining with mouse anti-human CD99 (Invitrogen, Waltham, MA, USA) (Figure S1). The cells were confirmed to be free of mycoplasma through IDEXX or with MycoStrip™—Mycoplasma Detection Kit (Invivogen, San Diego, CA, USA). The cells were used for experiments at passage ≤ 20. The characteristics of each patient tumor used are shown in Figure 1A.

### 2.2. Sequencing of Patient Tumors

For sample collection, all the subjects underwent a standard-of-care scheduled surgical resection and/or diagnostic biopsy procedure in which fresh tumor samples and a blood sample were collected. The fresh tumor samples were flash-frozen in dry ice and shipped to either Ashion Analytics, LLC (Ashion, Phoenix, AZ, USA), or Sema4 Genomics, both CLIA-certified laboratories. At Ashion, RNA extraction was performed via Qiagen AllPrep (Germantown, MD, USA), and libraries were generated with KAPA RNA RiboErase (Roche LifeSciences (Indianapolis, IN, USA). The libraries were then clustered on flow cells and sequenced using the NovaSeq 6000 (Illumina, San Diego, CA, USA). At Sema4, RNA was extracted followed by the quality/quantity assessment of the nucleic acids. Libraries were prepared using TruSeq^®^ RNA exome (Illumina, San Diego, CA, USA) and/or TruSeq^®^ Stranded Total RNA Gold (Illumina). The libraries were sequenced using the NovaSeq 6000.

A gene expression analysis was performed as previously described [23]. A previously published standardized Z score method was used to determine statistically significant differences in each sample, relative to a whole-body reference of 22 normal tissue gene expression levels [7]. A heatmap was generated from NRZ scores using the Complex Heatmap R package version 2.13.1 [24] (Figure 1B).

### 2.3. IC50 Values and Genetic Correlation

A total of 5000 cells were seeded on a 96-well plate overnight and treated with drugs for 48 h. Drug concentrations ranged from 0 nM to 3125 nM for Panobinostat (Selleck, Houston, TX, USA), and 0 nM–12,500 nM for Ceritinib (Cayman Chemical Company, Ann Arbor, MI, USA), Palbociclib (Cayman Chemical Company), and Ruxolitinib (Selleck). CellTiter-Glo Luminescent Cell Viability Assay (Promega, Madison, WI, USA) was used to determine cell viability after treatment following the manufacturer’s protocol; luminescence for each drug-treated well was normalized to vehicle-treated controls and GraphPad Prism was used to calculate the IC50 values. Each experiment was performed in triplicate and replicated for 3 independent experiments. Pearson correlation coefficients between IC50 values and gene expression were calculated using GraphPad Prism 9.2.0.

### 2.4. RNA Sequencing

An RNA expression analysis was performed on vehicle-treated and Panobinostat-treated SL00755 and SL01258 cells. A total of 5 × 10^5^ cells were seeded in T25 flasks and incubated overnight. The cells were then treated with vehicle or Panobinostat at their approximate IC50 and ½ IC50 concentrations. After incubation with the drug for 12 or 24 h, the cells were collected and centrifuged into pellets. The pellets were then rinsed with 1000 µL cold PBS, and then rinsed twice with 500 µL cold PBS containing 1 µL SuperaseIn RNase inhibitor (Invitrogen). The pellets were then resuspended in 250 µL cold PBS containing 2 µL SuperaseIn and stored at −80 °C.

Transcriptome profiling was performed by RNA sequencing. Total RNA was isolated using miRNeasy Mini Kit (Qiagen, Germantown, MD, USA) per the manufacturer’s instructions. RNA integrity was evaluated by an Agilent 2100 Bioanalyzer profile (Agilent, Santa Clara, CA, USA). Ribosomal RNA was selectively depleted by employing RiboZero Globin (Illumina, San Diego, CA, USA). Next-generation sequencing libraries were prepared using Illumina TruSeq Stranded Total RNA kits (Illumina) as directed. Briefly, the RNA was fragmented using divalent cations under elevated temperature following purification. Cleaved RNA fragments were copied into first-strand cDNA using reverse transcriptase and random primers. Strand specificity was achieved by replacing dTTP with dUTP during second-strand cDNA synthesis. The products were PCR-amplified and purified to create the final cDNA library which was sequenced with 100 bp paired-end reads. Read mapping was performed using STAR in BaseSpace (Illumina). DESeq2 was used for normalization and differential expression analysis. The experiments were performed in triplicate and pathway changes and pathway diagrams were generated using QIAGEN IPA (QIAGEN Inc., https://digitalinsights.qiagen.com/IPA (accessed on 30 June 2022)) [25].

### 2.5. Synergy Treatment and Analysis

Cells were seeded in 96-well plates with 5000 cells per well and incubated overnight. The cells were then treated with Doxorubicin (Cayman Chemical Company) (0, 62.5, 125, or 250 nM), Etoposide (Selleck) (0, 15, 30, or 60 µM), and/or Panobinostat (0, 12.5, 25, 50, or 100 nM) for 48 h. CellTiter-Glo Luminescent Cell Viability Assay (Promega) was used to assess cell viability following the manufacturer’s protocol. Each experiment was performed in triplicate and replicated for 3 independent experiments. Synergy was assessed using SynergyFinder 3.0 with the Bliss independence model.

### 2.6. Chemotherapy Combination Treatment and Analysis

Cells were seeded in 96-well plates with 5000 cells per well and incubated overnight. The cells were then treated with Panobinostat (0, 30, 50, and 80 nM) and/or VDC: Vincristine (20 nM, Selleck), Doxorubicin (100 nM, Cayman Chemical Company), Cyclophosphamide (100µM, Selleck); or IE: Ifosfamide (400µM, Selleck), Etoposide (30µM, Selleck) for 48 h. CellTiter-Glo Luminescent Cell Viability Assay (Promega) was used to assess cell viability following the manufacturer’s protocol. Each experiment was performed in triplicate and replicated for 3 independent experiments. Graphpad Prism was used for all the statistical analyses.

### 2.7. Western Blotting and Densitometry

In total, 200,000 cells were seeded per well in a 6-well plate overnight and treated with drug(s) for 24 and/or 48 h. The cells were treated with the vehicle, approximate IC50, or ½ IC50 of Panobinostat for the single agent experiment, and treated with vehicle, 50 nM Panobinostat, 125 nM Doxorubicin, or Panobinostat and Doxorubicin for the synergy experiment. The cells were lysed in RIPA buffer containing protease and phosphatase inhibitors (Thermo Scientific, Waltham, MA, USA). Protein concentrations were determined by Pierce™ BCA Protein Assay Kit (Thermo Scientific). Lysates were denatured at 95 °C for 5 min and subjected to electrophoresis on a 4–12% Bis-Tris gel (25 µg of protein were loaded per lane). Gels were transferred to a nitrocellulose membrane using the iBlot 2 Dry blotting system (Invitrogen). Protein expression was detected using the Radiance Plus or Radiance Q chemiluminescent substrate (Azure Biosystems, Dublin, CA, USA) and imaged using the Azure 600 Imaging System (Azure Biosystems). Signal intensities were quantified using the AzureSpot software 2.2.167 to select bands and subtract background. Intensities were normalized to ß-Actin, and fold changes were calculated for each drug-treated sample over the vehicle-treated one; GraphPad Prism was used for statistical analysis. All the experiments are representative of 3 independent assays. The primary antibodies used were rabbit monoclonal phospho-histone H2AX (#9718), rabbit polyclonal histone H2AX (#2595), rabbit monoclonal phospho-CHK2 (#2197), rabbit monoclonal CHK2 (#6334), mouse monoclonal CHK1 (#2360), rabbit polyclonal cleaved Caspase 3 (#9661), rabbit polyclonal phospho-Rb (#9308), mouse monoclonal Rb (#9309), rabbit polyclonal ß-Actin (#4967), mouse monoclonal ß-Actin (#3700) (Cell Signaling, Danvers, MA, USA), and mouse monoclonal Cyclin D1 (05-362) (Millipore Sigma, Burlington, MA, USA). The secondary antibodies used were HRP-linked anti-rabbit IgG (#7074) and HRP-linked anti-mouse IgG (#7076) (Cell Signaling).

### 2.8. Cell Cycle Experiment/Analysis

EWS cell lines were transduced with Incucyte^®^ Cell Cycle Green/Red Lentivirus Reagent (Sartorius Cat. #4779, Göttingen, Germany). For transduction, the cells were seeded in a 6-well plate overnight, the lentivirus was then added at a multiplicity of infection (MOI) of 1 with 0.8 µg/mL polybrene in complete media. A total of 24 h after the lentivirus addition, the lentivirus-containing media was removed and replaced with complete culture media. A total of 48 h after the lentivirus removal, puromycin was used to select for transduced cells. The cells were cultured in puromycin-containing media for 5–10 days to ensure selection.

For cell cycle analysis, 3000 cells (stable cell line expressing Incucyte^®^ Cell Cycle Green/Red Lentivirus Reagent) were seeded in 96-well plates and incubated overnight. The cells were treated with the vehicle, approximate IC50, or ½ IC50 of Panobinostat for 24 h. Upon drug treatment, the plates were placed in an Incucyte^®^ S3 for live cell imaging. Images were quantified using the Incucyte^®^ 2022A Basic Anlayzer software. The percentage of cells within each phase was calculated as (# counted cells within specified phase/# total cells counted in any phase ×100). Graphpad Prism was used for all the statistical analyses.

## 3. Results

### 3.1. HDAC2 Is Overexpressed in EWS Tumors and Cells Are Sensitive to HDAC Inhibition

Full exome and transcriptome analyses were performed on ten EWS patient samples and a heat map was generated to compare the RNA expression levels of the genes of interest; expression levels are compared to a whole-body reference of 22 normal tissue gene expression levels [7] (Figure 1B). Gene targets were chosen based on expression levels across the patient samples and the ability to target these pathways with available chemotherapy agents. The overexpression of *HDAC2* (average Z score = 2.40; ranging from 1.39 to 4.17), *ALK* (average Z score = 1.72; ranging from 0.51 to 3.19), *CDK4* (average Z score = 2.06; ranging from −0.42 to 3.06), and *JAK1* (average Z score = 4.32; ranging from −0.95 to 7.03) was identified in most samples. It is hypothesized that these overexpressed pathways may be contributing to tumor growth and survival. The following agents were chosen to test in vitro: Panobinostat, pan-HDAC inhibitor; Ceritinib, ALK inhibitor; Palbociclib, CDK4/6 inhibitor; and Ruxolitinib, JAK inhibitor.

**Figure 2:**
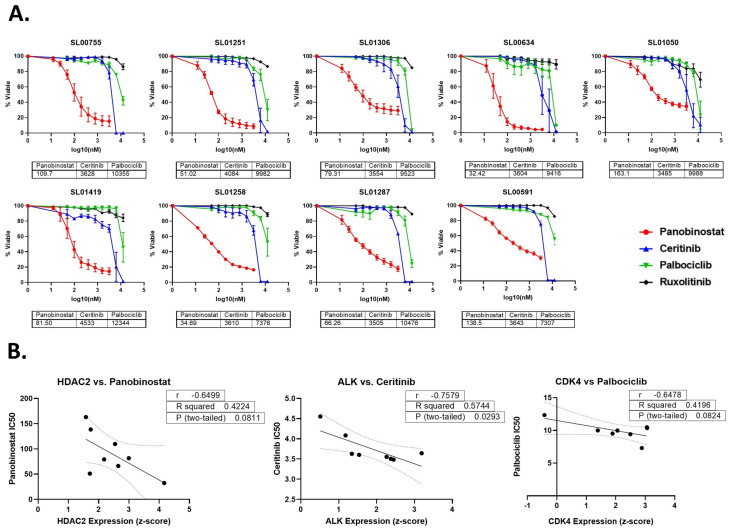
Ewing sarcoma cells are sensitive to Panobinostat. (**A**) Ewing sarcoma patient-derived cell lines were treated with targeted agents (Panobinostat, Ceritinib, Palbociclib, or Ruxolitinib) for 48 h. Viability was measured with CellTiter-Glo 2.0 and IC50s were calculated using the Absolute IC50 function in GraphPad Prism. (**B**) The IC50 values (nM) of the targeted agents were correlated to HDAC2 (Panobinostat), ALK (Ceritinib), or CDK4 (Palbociclib) RNA expression using the correlation analysis in GraphPad Prism. The solid line indicates the line of best fit and the dotted line represents the 95% confidence interval.

In total, 9 EWS patient-derived cell lines were treated with the chosen targeted agents, and cell viability was measured after 48 h of treatment (Figure 2A). Panobinostat was the most effective of all the agents with clinically relevant IC50 values. The cell lines were sensitive to Ceritinib and Palbociclib at higher concentrations and the IC50 values were above clinical relevance. There was no sensitivity to Ruxolitinib at any of the clinically relevant concentrations tested; therefore, the IC50 values were not determined. RNA expression was analyzed and revealed a trend between higher HDAC2 expression and lower IC50 values (r = −0.6499), suggesting that the cell lines from patient tumors that had higher HDAC2 expression are more sensitive to treatment with Panobinostat (Figure 2B). A trend between Palbociclib sensitivity and CDK4 expression was also observed (r = −0.6478). Of note, a significant correlation does exist between ALK expression and Certinib, but the IC50 values were above clinical relevance (r = −0.7579, *p* < 0.05).

### 3.2. Panobinostat Decreases Cell Cycle and DNA Damage Repair Pathways

**Figure 3:**
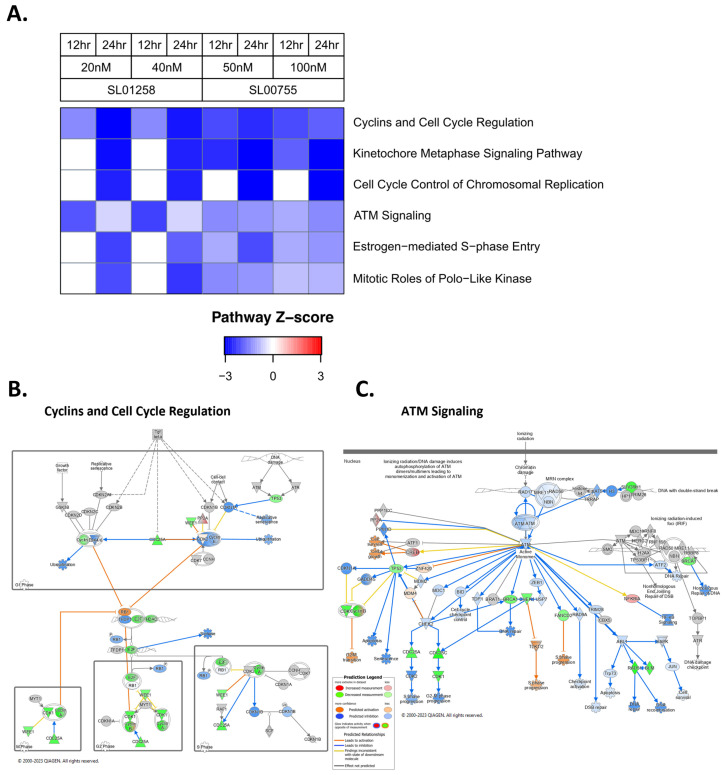
Panobinostat decreases the RNA expression of cell cycle and DNA damage-related pathways. (**A**–**C**) Ewing sarcoma patient-derived cell lines (SL00755 and SL01258) were treated with Panobinostat (vehicle control, approximate ½ IC50, or approximate IC50) for 12 or 24 h. RNA sequencing was performed and ingenuity pathway analysis (IPA) was used to identify significant changes in the pathways of interest by comparing the treated samples to the vehicle-treated controls (**A**). IPA was used to generate diagrams of indicated pathways using SL00755 treated with 50 nM Panobinostat for 24 h as the reference dataset (**B**,**C**).

Since there was a sensitivity of all the cell lines tested to Panobinostat, RNA sequencing was used to analyze changes in RNA expression in the Panobinostat-treated cells compared to the vehicle-treated cells to identify pathway changes induced by the treatment (Figure 3A). The two cell lines tested used for RNA expression analysis treatment included SL00755 and SL01258, which had a range of IC50s of 109.7 nM and 34.69 nM, respectively (Figure 2A). The Cyclins and Cell Cycle Regulation pathway was downregulated after treatment, as well as the other pathways related to cell cycle progression such as Cell Cycle Control of Chromosomal Replication, Estrogen-mediated S-phase Entry, Kinetochore Metaphase Signaling, and Mitotic Roles of Polo-Like Kinases, indicating that normal cell cycle progression was not occurring. An ingenuity pathway analysis (IPA) of the two cell lines tested, SL00755 and SL01258, further highlighted specific genes and cellular processes that are decreased or predicted to be inhibited by the Panobinostat treatment (Figure 3B,C and Figure S3A–C). The Cyclins and Cell Cycle Regulation pathways show that the E2F transcription factors are decreased and therefore the S-phase of the cell cycle is predicted to be inhibited. Additionally, CCND1 (encodes Cyclin D1) was decreased (−2.02444 log2FoldChange), and the CDK4/6 complex was predicted to be inhibited. These proteins are involved in the G1 phase of the cell cycle, so these data indicate that Panobinostat may be preventing cell cycle progression in the G1 phase, which is consistent with the prediction of S-phase inhibition. The ATM signaling pathway is decreased, indicating that S-phase progression, G2-M phase progression, cell survival, and DNA repair are all predicted to be inhibited by the Panobinostat treatment. The Cell Cycle Control of Chromosomal Replication, Estrogen-mediated S-phase Entry, and Mitotic Roles of Polo-Like Kinases pathways consistently show the inhibition of cell cycle progression, decreases in CCND1, the inhibition of RB1 phosphorylation, decreases in E2F transcription factors, and the inhibition of CDK4/6 complex (Figure S2A–C). The Kinetochore Metaphase Signaling pathway shows that AURKB, which is one of the central proteins of this pathway, is decreased (Figure S3D).

**Figure 4:**
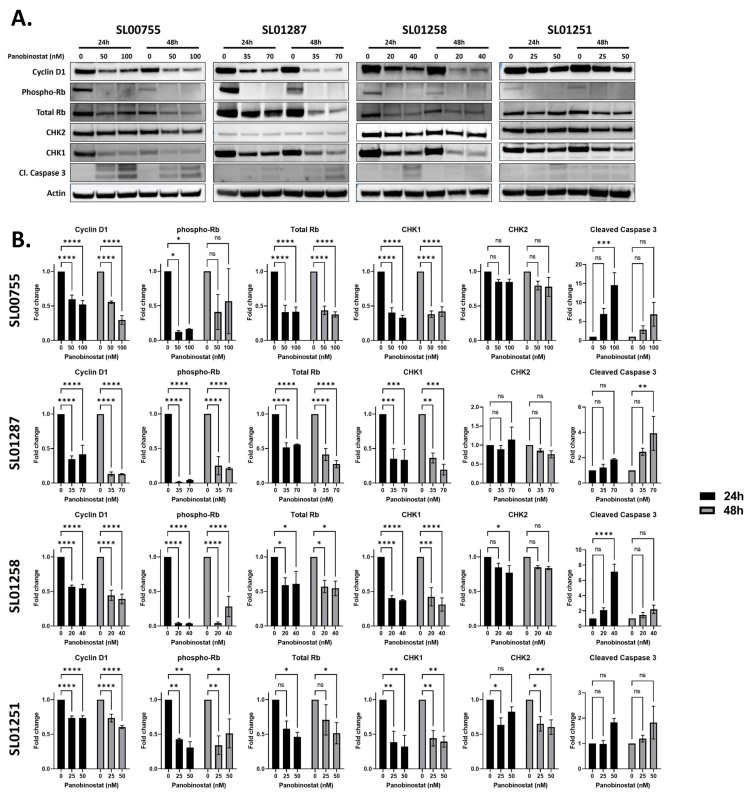
Panobinostat decreases the expression of proteins involved in the cell cycle and DNA damage response. (**A**,**B**) Ewing sarcoma patient-derived cell lines were treated with Panobinostat (vehicle control, approximate ½ IC50, or approximate IC50) for 24 or 48 h, and protein expression was determined by Western blotting with the indicated antibodies (**A**). Representative uncropped blots are shown in Figure S4. Signal intensities were quantified using the AzureSpot software to select bands and subtract background. Intensities were normalized to ß-Actin, and fold changes were calculated for each drug-treated sample over vehicle-treated one. Two-way ANOVA with Dunnett’s multiple comparisons test indicates significance: ns: no significance, * *p* < 0.05, ** *p* < 0.01, *** *p* < 0.001, and **** *p* < 0.0001 (**B**).

Given that the RNA sequencing analysis revealed that Panobinostat leads to a decrease in cell cycle and DNA damage repair-associated pathways, we further investigated this on the protein level. Cyclin D1, in complex with CDK4/6, leads to the phosphorylation of Retinoblastoma protein (Rb). This phosphorylation of Rb results in its dissociation from E2F family transcription factors; these transcription factors are then able to activate many genes required for progression to the S phase [26]. When EWS cells (SL00755, SL01287, SL01258, and SL01251) were treated with Panobinostat, the expression of Cyclin D1, total Rb, and phospho-Rb were significantly repressed (0.13–0.60 fold change at IC50 concentration at 48 h, *p* ≤ 0.001 at 24 and 48 h; 0.27–0.54 fold change at IC50 concentration at 48 h, *p* ≤ 0.05 at 24 and 48 h; and 0.1–0.5 fold change, *p* ≤ 0.05 at 24 h, respectively) for all the cell lines tested (Figure 4A,B), consistent with our RNA sequencing data, suggesting the inhibition of cell cycle progression.

CHK1 and CHK2 are involved in the DNA damage repair of single-stranded and double-stranded breaks, respectively [27]. The treatment of EWS cells resulted in a significant decrease in CHK1 expression and a slight decrease in CHK2 expression, though not significant (Figure 4A,B). This These data are consistent with what we observed at the RNA level. The ATM signaling pathway shows that CHK1 expression was decreased on the RNA level (Figure 3), and we observed a significant decrease in expression in all the cell lines on the protein level (0.19–0.42 fold change, *p* ≤ 0.01 at 48 h) (Figure 4). CHK2 was predicted to be inhibited, though decreased expression was only observed in two cell lines, SL01258 and SL01251 (0.77 fold change, *p* ≤ 0.05 at 40 nM for 24 h for SL01258; 0.60 fold change *p* ≤ 0.05 at 50 nM for 48 h for SL01251). Additionally, the treatment of Panobinostat resulted in an increase in Caspase 3 cleavage (14.5 fold change, *p* ≤ 0.001 at 100 nM for 24 h for SL00755; 3.9 fold change, *p* ≤ 0.01 at 70 nM for 48 h for SL00755; 7.1 fold change, *p* ≤ 0.0001 at 40 nM for 24 h for SL01258), indicating apoptosis (Figure 4A,B).

### 3.3. Panobinostat Leads to a G1 Cell Cycle Arrest

**Figure 5:**
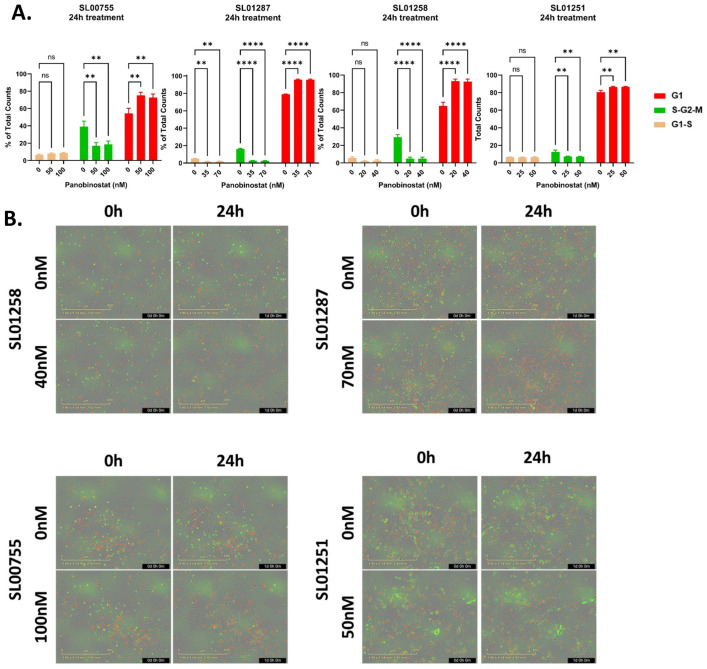
Panobinostat induces G1 cell cycle arrest in Ewing Sarcoma. (**A**,**B**) Cells stably expressing the Incucyte^®^ Cell Cycle Lentivirus Reagent were treated with Panobinostat (vehicle control, approximate ½ IC50, or approximate IC50) for 24 h and images were collected on an Incucyte S3 live cell imaging system. (**A**) Images were quantified using the Incucyte^®^ Basic Anlayzer software and the percentage of cells, out of all the cells counted within that well, in each phase are shown. Two-way ANOVA with Dunnett’s multiple comparisons test indicates significance: ns: no significance, ** *p* < 0.01, and **** *p* < 0.0001. The data are the average of 3 experiments and the error bars are SEM. (**B**) The representative images of the indicated timepoint and Panobinostat concentrations are shown for each cell line tested. The cells in the G1 phase are shown in red, the cells in G1/S are shown in yellow, and the cells in S/G2/M are shown in green.

Based on the repression of the proteins involved in cell cycle progression, we hypothesized Panobinostat treatment would lead to cell cycle arrest in EWS cells. After 24 h of treatment, there was a significant increase in cells in the G1 phase and a significant decrease in cells in the S/G2/M phase, indicating G1 cell cycle arrest (Figure 5). Taken together, these data indicate that Panobinostat represses the expression of the proteins involved in DNA damage repair and cell cycle progression which leads to G1 cell cycle arrest. This confirms our RNA sequencing data with which IPA predicted the inhibition of both G2/M and S-phase progression by the Panobinostat treatment.

### 3.4. Panobinostat Synergizes with the Standard of Care Chemotherapeutic Agents, Doxorubicin and Etoposide, in EWS Cells

**Figure 6:**
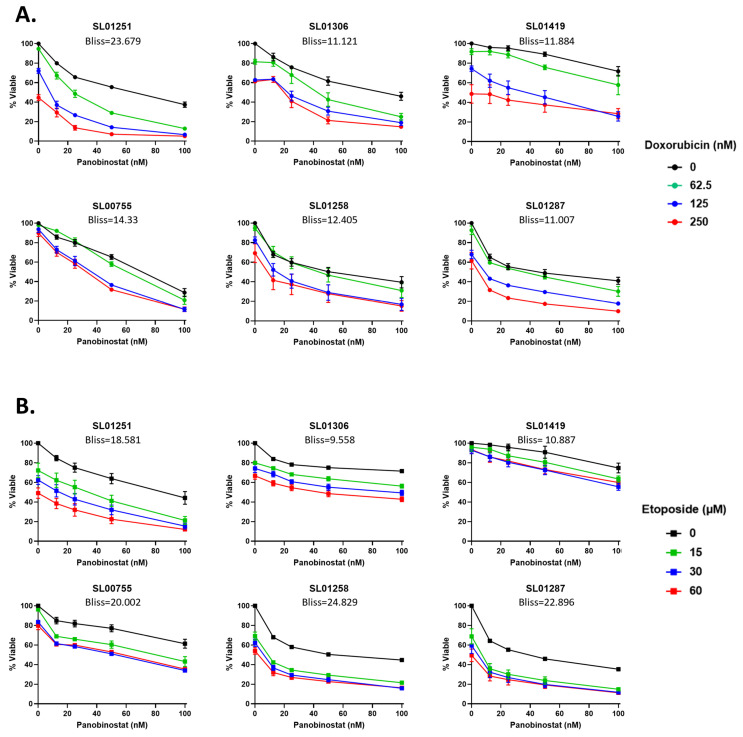
Panobinostat synergizes with standard-of-care chemotherapeutics. (**A**) Ewing sarcoma patient cells were treated with Panobinostat (0, 12.5,25, 50, or 100 nM) alone or in combination with (**A**) Doxorubicin (0, 62.5, 125, or 250 nM) or (**B**) Etoposide (0, 15, 30, or 60 µM) for 48 h. Cell viability was measured using CellTiter-Glo 2.0; the viability of the drug-treated cells was normalized to the vehicle-treated controls. The data are the average of ≥3 experiments and the error bars are SEM. Bliss synergy scores were calculated using SynergyFinder. Bliss scores of −10 to 10 indicate an additive effect; scores of greater than 10 indicate a synergistic effect.

Panobinostat decreases the activation of the ATM pathway (Figure 3), resulting in the decreased expression of proteins involved in DNA damage repair (Figure 4). Therefore, we hypothesized that combining Panobinostat with DNA-damaging agents would lead to a synergistic effect on cell death. Doxorubicin and Etoposide are chemotherapeutic drugs used in the upfront treatment of EWS patients [3] and are known to induce DNA damage [28,29]. When Panobinostat is combined in vitro with either of these drugs at clinically relevant doses [30], there is a synergistic response, evidenced by a Bliss synergy score of greater than 10 in all the cell lines, with the exception of SL01306 which had a Bliss score of 9.558 when treated with the combination of Panobinostat and Etoposide; this is classified as an additive effect, though approaching a synergistic Bliss score (Figure 6A,B).

### 3.5. The Combination of Standard of Care Chemotherapeutic Agents with Panobinostat Leads to an Accumulation of DNA Damage and a Decrease in DNA Damage Repair

**Figure 7:**
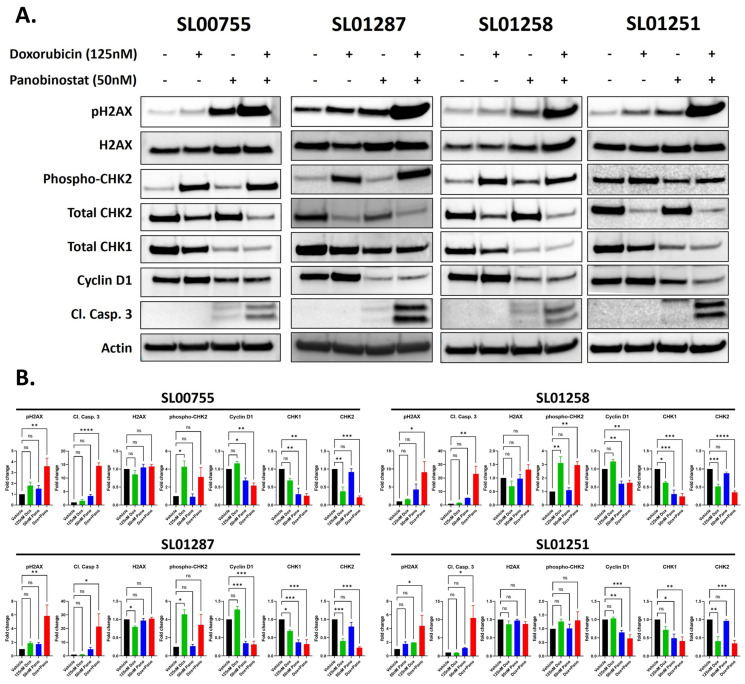
The combination of Panobinostat and Doxorubicin increases the expression of DNA damage and apoptosis markers and decreases the expression of DNA damage response proteins. (**A**,**B**) Ewing sarcoma patient cells were treated with Panobinostat (50 nM) alone or in combination with Doxorubicin (125 nM) for 24 h and protein expression was determined by Western blotting with the indicated antibodies (**A**). Representative uncropped blots are shown in Figure S4. Signal intensities were quantified using the AzureSpot software to select bands and subtract background. Intensities were normalized to ß-Actin, and fold changes were calculated for each drug-treated sample over vehicle-treated ones. One-way ANOVA with Dunnett’s multiple comparisons test indicates significance: ns: no significance, * *p* < 0.05, ** *p* < 0.01, *** *p* < 0.001, and **** *p* < 0.0001 (**B**).

The observation that Panobinostat synergizes with Doxorubicin and Etoposide led us to investigate the possible mechanism(s) of synergy. We have shown that Panobinostat leads to a decrease in DNA damage response proteins (Figure 4) and therefore we hypothesized that when paired with a DNA damaging agent the combination would lead to an accumulation of DNA damage. Because Doxorubicin and Etoposide are both Topoisomerase II inhibitors and are known to induce DNA damage, we hypothesized that the combinations with Panobinostat would lead to the accumulation of DNA damage and that this could be responsible for the observed synergistic response. To test this hypothesis, Doxorubicin- and/or Panobinostat-treated cells were evaluated at the protein level for changes in DNA damage response and DNA damage accumulation. Doxorubicin or Panobinostat treatment alone led to slight, though not significant, increases in H2AX phosphorylation, a marker of DNA damage. The combination treatment, at clinically relevant concentrations, led to a significant increase in DNA damage compared to the vehicle-treated cells, indicated by increases in H2AX phosphorylation (5.72 average fold change, *p* ≤ 0.05) in each of the four cell lines tested (Figure 7). Doxorubicin alone led to a significant increase in the phosphorylation of CHK2 (3.97 average fold change in SL00755, SL01287, SL01258, *p* ≤ 0.05), which is known to be downstream of ATM in response to DNA damage [31], in SL00755, SL01287, and SL01258. In SL00755 and SL01287, the combination treatment led to an increase in phospho-CHK2, though not significant when compared to vehicle, and decreased compared to Doxorubicin alone. Treatment with Doxorubicin alone led to a significant decrease in CHK2 (0.43 average fold change, *p* < 0.001) and treatment with Panobinostat alone led to a significant decrease in CHK1 (0.32 average fold change, *p* ≤ 0.05) in all the cell lines tested. When the two drugs were combined, there was a significant decrease in both CHK1 and CHK2 (0.31 average fold change, *p* < 0.01; and 0.285 average fold change, *p* < 0.001, respectively) in all the cell lines tested; suggesting that a possible mechanism of the synergy observed between Panobinostat and Doxorubicin is the decrease in DNA damage response proteins and the accumulation of DNA damage.

### 3.6. Panobinostat Increases the Effect of Standard-of-Care Chemotherapy Combinations

**Figure 8:**
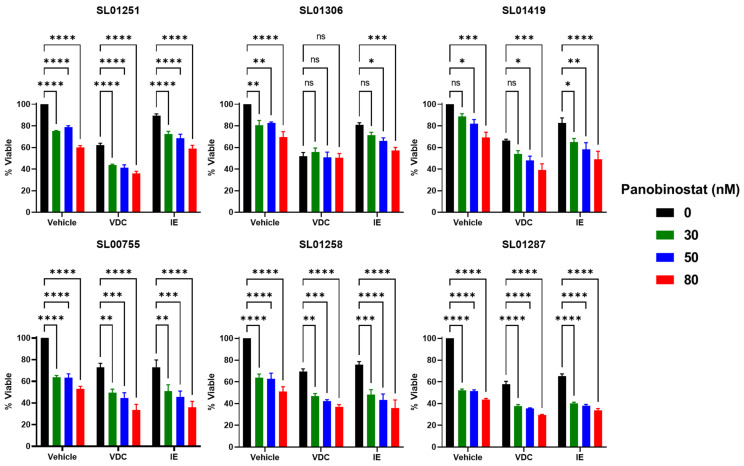
Panobinostat increases the effect of standard-of-care chemotherapy. Ewing sarcoma patient cells were treated with Panobinostat, the standard of care chemotherapy combinations, or a combination of Panobinostat with chemotherapy. Cell viability was measured following 48 h of treatment using CellTiterGlo 2.0; viability of the drug-treated cells was normalized to the vehicle-only treated controls. The data are the average of ≥3 experiments and the error bars are SEM. Two-way ANOVA with Dunnett’s multiple comparisons test indicates significance: ns: no significance, * *p* < 0.05, ** *p* < 0.01, *** *p* < 0.001, and **** *p* < 0.0001. Drug concentrations: VDC—20 nM Vincristine, 100 nM Doxorubicin, 100 µM Cyclophosphamide; IE—400 µM Ifosfamide, 30 µM Etoposide.

Given that Panobinostat synergizes with both Doxorubicin and Etoposide, we hypothesized that the addition of Panobinostat would significantly increase the efficacy of standard-of-care chemotherapy combinations compared to that of the chemotherapy alone. Standard-of-care treatment consists of alternating cycles of VDC and IE [3]; when Panobinostat was combined with each of these combinations, viability was significantly reduced compared to that of the chemotherapy combination alone (Figure 8). These data suggest that the addition of Panobinostat to the standard-of-care treatment may lead to improved patient response.

## 4. Discussion

Histone deacetylases (HDACs) are known to play a significant role in tumorigenesis. Among them, class 1 HDACs specifically promote cell proliferation and inhibit apoptosis [19]. As such, histone deacetylase inhibitors (HDACis) have been studied and developed for cancer treatment. In EWS patients, high levels of individual class 1 HDAC expression have been associated with decreased overall survival. The effects of various HDAC inhibitors have been studied in EWS cells [32]. Vorinostat, a pan-HDACi, has been studied in combination with irinotecan and temozolomide, the standard medications used for relapsed EWS. Sequential administration after chemotherapy has been shown to increase the levels of DNA damage and apoptotic cells. This effect was suggested to be dependent on the STAT3/AKT/MAPK and p53 pathways [33]. Additionally, the HDAC inhibitors Entinostat and Romidepsin have been shown to synergize with Doxorubicin in vitro, and inhibit tumor growth when combined with an embryonic ectoderm development (EED, a protein which is part of the PRC2 complex) inhibitor in vivo. This synergy with Doxorubicin was attributed to a pro-apoptotic gene signature, AKT signaling, and ubiquitin-mediated protein degradation [32]. However, the effects of this combination on DNA damage and repair were not investigated. Based on our RNA sequencing and protein expression data which indicate a decrease in DNA damage repair by Panobinostat, we believe that a possible mechanism of the synergy observed between pan-HDACi Panobinostat, and Doxorubicin or Etoposide could be due to a decrease in DNA damage repair and the accumulation of DNA damage, though further experiments investigating DNA damage repair would be needed to confirm this.

Panobinostat is an FDA-approved pan-HDACi that has been studied across cancers and is commonly used in the treatment of multiple myeloma [34,35]. Panobinostat has been shown to increase DNA double-stranded breaks and apoptosis in both AML and neuroblastoma and block the S and G2/M cell cycle checkpoints in AML [36,37]. Checkpoint kinase 1 (CHK1) and Checkpoint kinase 2 (CHK2) are involved in the pathways that repair DNA damage and can lead to G2/M and S arrest, respectively [38]. CHK1 is a serine/threonine kinase that is primarily initiated by single-stranded breaks and is responsible for initiating cell cycle arrest, allowing for DNA repair and cell survival. CHK2 is initiated by factors that induce double-strand breaks, such as ionizing radiation or chemotherapeutic agents. If the CHK1 and CHK2 pathways are blocked or not able to repair the DNA damage, the cell will go into apoptosis [31]. Panobinostat was previously shown to suppress the expression of CHK1 which improved the cytarabine and daunorubicin sensitivities in AML cells, and this combination led to an increase in survival in a xenograft model [36]. HDAC inhibition by Panobinostat has also been shown to downregulate the CHK1 pathway in neuroblastoma. This downregulation led to the synergistic cytotoxicity, apoptosis, and abrogation of cell cycle arrest with the DNA damaging agents Doxorubicin and Etoposide given simultaneously in neuroblastoma [37]. These studies are consistent with what we have observed in EWS; though there are other suggested mechanisms of synergy between HDAC inhibitors and chemotherapeutic agents which we have not fully investigated in the context of EWS cells [32,33]. Future experiments would aim to confirm a mechanism, or mechanisms, of synergy between Panobinostat and chemotherapeutic agents.

Through further investigation of combining Panobinostat with standard-of-care drugs, we identified that, not only does it synergize with chemotherapeutics, but the addition of Panobinostat to the upfront standard-of-care chemotherapy combinations significantly reduces cell viability compared to that of the chemotherapy combinations alone. This would suggest that the addition of Panobinostat to upfront treatment may lead to better efficacy of the treatment and improve patient outcomes. There has been little improvement in treatment options and survival rates for EWS patients in many years, highlighting a need for agents that are effective against this disease. There have been more recent studies investigating alternate treatment options including the use of nano-technology, antibody therapy, and combinations of targeted agents which may provide some hope that new treatment options will be available sooner rather than later [39,40,41]. Our study is limited in that it does not address the in vivo efficacy or toxicity of this drug combination; future experiments would aim to investigate the in vivo efficacy of HDAC inhibition combined with chemotherapy. We believe that Panobinostat may be another option in which we can more effectively target EWS cells and improve the efficacy of our current standard of care treatment.

In addition to targeting HDACs, we believe Panobinostat is targeting multiple genes/proteins that are overexpressed in many patient samples and have been shown to be important in EWS cell proliferation and survival. Cyclin D1 (encoded by *CCND1*) and CDK4 (encoded by *CDK4*) have been shown to be required by EWS cells for survival [42] and we have shown here that many of the patient samples sequenced overexpress these genes (average Z-scores = 3.51 and 2.06, respectively) (Figure 1). Interestingly, Panobinostat treatment leads to a decrease in Cyclin D1 gene and protein expression, and the CDK4/6 complex is predicted by IPA to be inhibited based on our RNA sequencing data. CHK1 is also overexpressed in many patient samples (average Z-score = 4.71, Figure 1B) and is known to promote tumor growth and confer resistance to chemotherapy, indicating that it may be playing a role in EWS tumor progression and survival [43,44,45]. We have shown here that Panobinostat treatment decreases the expression of CHK1 at both the gene and protein levels (Figure 3C and Figure 4).

The dysregulation of E2F family transcription factors is also known to promote cancer [46,47] and it has been reported that in EWS, the transcription of EWSR1/FLI1 target genes is partially mediated by E2Fs [48,49,50]. Our RNA sequencing data shows that the expression of E2F family members is decreased by Panobinostat (Figure 3B), again highlighting that Panobinostat targets multiple pathways which are known to be important in the context of EWS. Another important finding is that the expression of *AURKB* was decreased in response to Panobinostat. Aurora kinases, A and B, are involved in centrosome maturation and spindle formation, and chromosome segregation and cytokinesis, respectively. Both AURKA and AURKB are known to be upregulated in many cancers and their expression has been shown to be regulated by the EWSR1/FLI1 fusion [51]. Our patient tumor sequencing data shows the overexpression of AURKA and AURKB (average Z-score = 2.33 and 2.18, respectively); the result that Panobinostat decreases AURKB shows again that it is targeting another pathway which may be playing an important role in tumor progression. We believe that Panobinostat may be so effective because it targets several of these pathways which are known to be important in the progression or survival of EWS and/or cancer in general.

Our data show a trend between high HDAC2 expression and increased sensitivity to Panobinostat (low IC50 values), though not a significant correlation. This may suggest that patients with higher HDAC2 expression would have a better response to Panobinostat treatment, though we do not have a large enough sample size with a wide enough range of expression Z-scores yet to confidently say this. There is work being performed across different types of cancer to correlate expression data with drug sensitivity to better predict patient response to different treatment options using machine learning techniques based on historic patient data as well as cell line IC50 data [52,53]. We believe that in EWS HDAC2 expression may be a way to predict patient response to HDAC inhibitors.

## 5. Conclusions

Overall, our data indicate that HDAC2 is overexpressed in many EWS tumor samples and HDAC inhibition is effective in targeting EWS cells. Additionally, HDAC inhibition synergizes with standard-of-care chemotherapeutics and significantly increases the efficacy of standard-of-care chemotherapy combinations in EWS cells. Future experiments investigating the in vivo effects of HDAC inhibition on EWS xenograft models will provide further information on the potential efficacy of treating EWS patients with a combination of chemotherapeutics and an HDAC inhibitor. This work provides in vitro evidence that HDAC inhibition combined with standard of care may be an effective treatment option for EWS patients.

## Data Availability

The data presented in these studies are available upon request from the corresponding author.

## Supplementary Materials

The following supporting information can be downloaded at: https://www.mdpi.com/article/10.3390/cancers16213565/s1, Figure S1: CD99 expression of EWS patient-derived cell lines. Figure S2: RNA sequencing pathway analysis. Figure S3: Synergy analysis of Panobinostat and Doxorubicin or Etoposide; Figure S4: Full pictures of the Western blots.
