# Supplementary material for: Panobinostat Synergizes with Chemotherapeutic Agents and Improves Efficacy of Standard-of-Care Chemotherapy Combinations in Ewing Sarcoma Cells
Source: Cancers (Basel). 2024 Oct 23;16(21):3565. doi: 10.3390/cancers16213565 (PMC11545275; doi:10.3390/cancers16213565)
Supplement: Supplementary file 1 [file cancers-16-03565-s001.zip › Figures S1-S3.pdf]

Supplemental Figure S1.

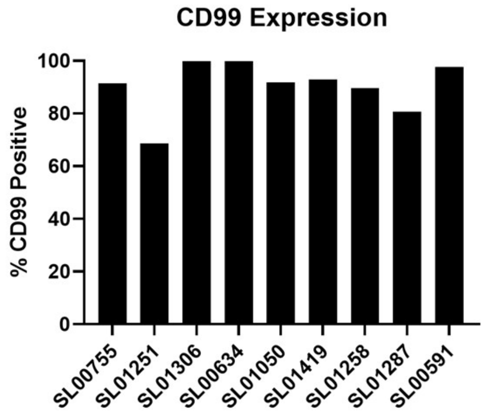

Figure S1. CD99 expression of EWS patient derived cell lines. Patient derived cell lines were stained with CD99 and % positivity was measured by flow cytometry to validate the cells as Ewing sarcoma.

Supplemental Figure S2.

A. Cell Cycle Control of Chromosomal Replication

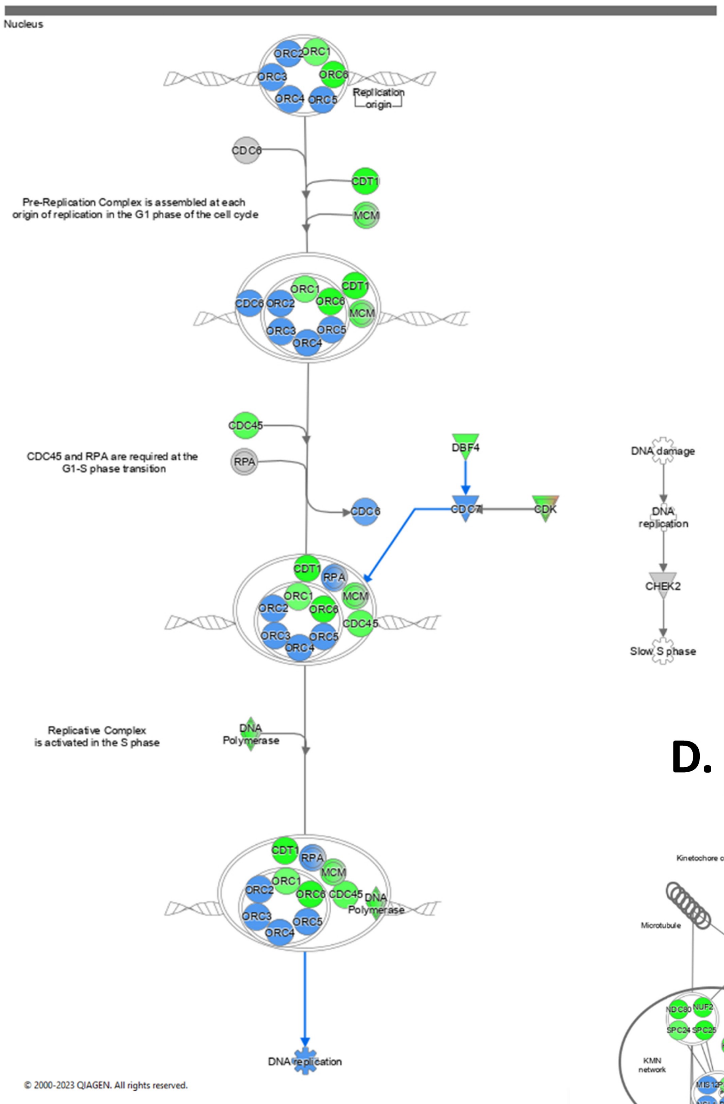

B. Estrogen-mediated S-phase Entry

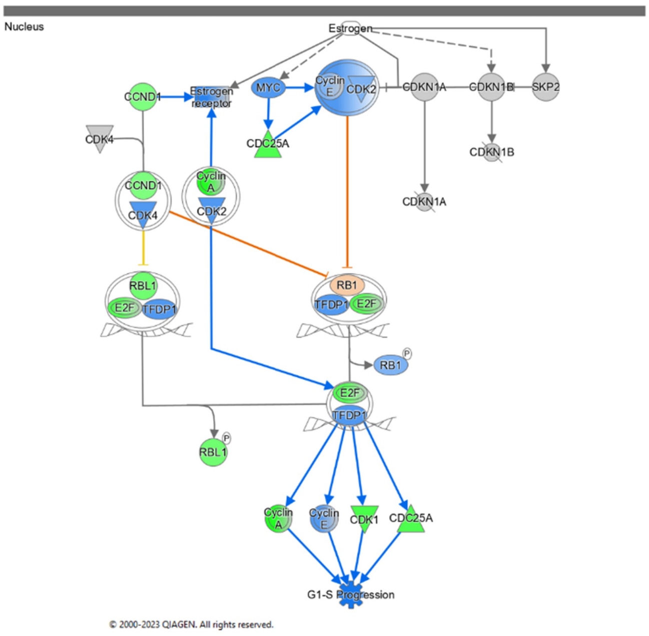

D. Kinetochore Metaphase Signaling Pathway

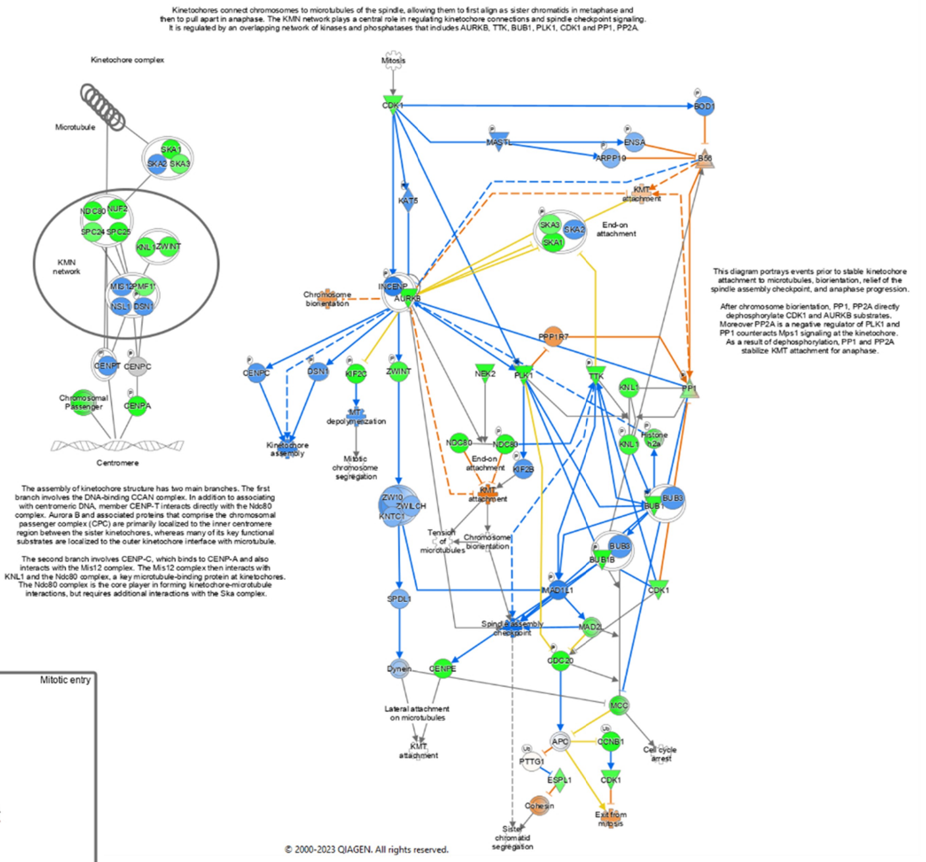

C. Mitotic Roles of Polo-Like Kinases

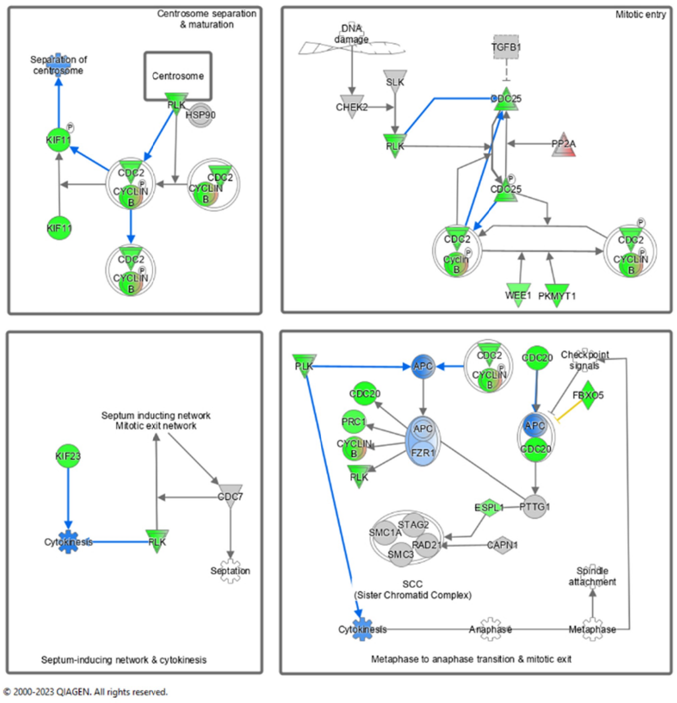

Figure S2. RNA sequencing pathway analysis.
Ingenuity Pathway Analysis (IPA) was used to generate diagrams of indicated pathways using SL00755 treated with 50nM Panobinostat for 24 hours as the reference dataset to show changes in gene expression.

Supplemental Figure S3.

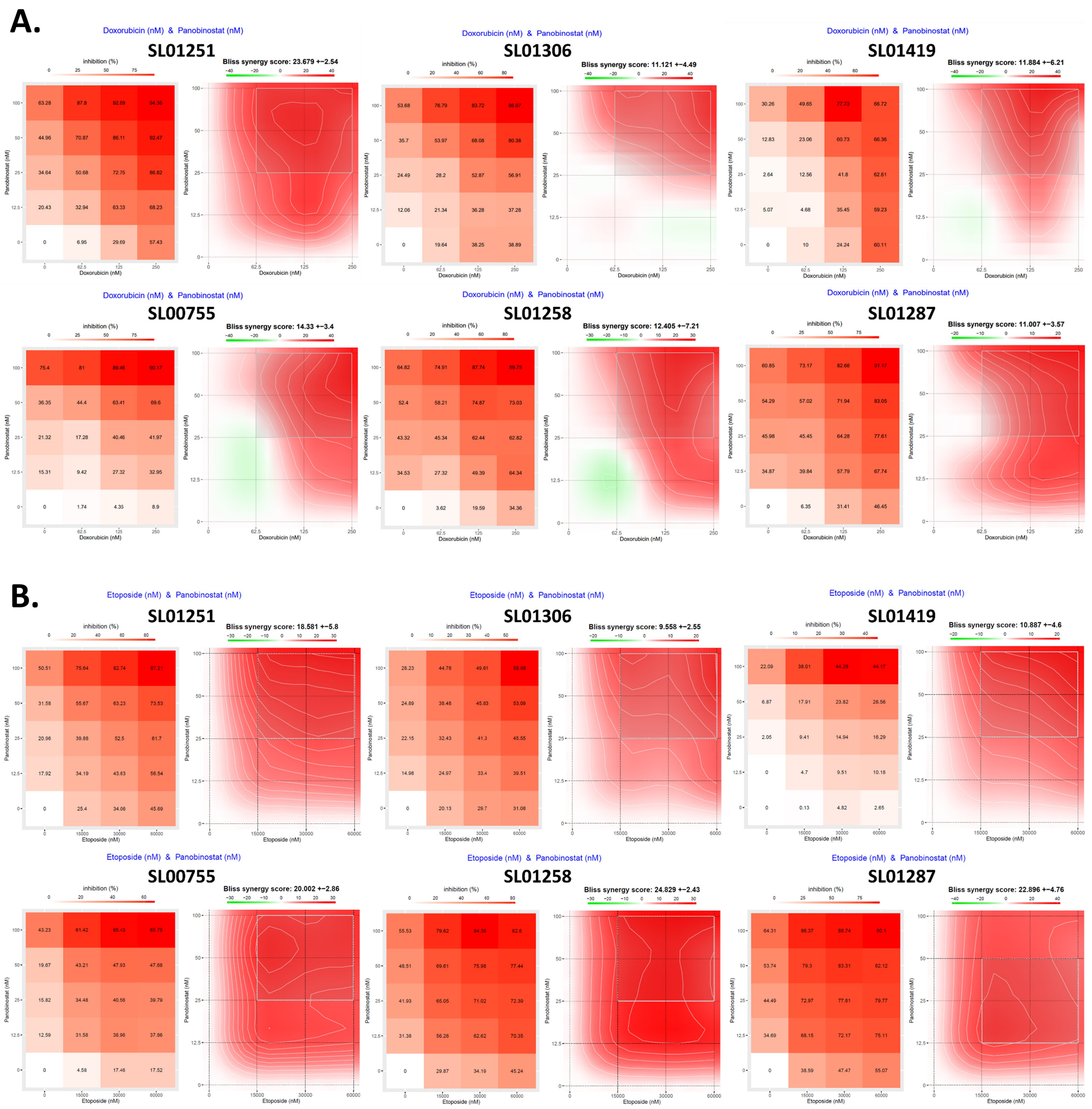

Figure S3. Synergy analysis of Panobinostat and Doxorubicin or Etoposide. SynergyFinder 3.0 was used to analyze response for synergy following drug treatments. Bliss scores of -10 to 10 indicate an additive effect; scores of greater than 10 indicate a synergistic effect.
