# Supplementary figures and images for: Panobinostat Synergizes with Chemotherapeutic Agents and Improves Efficacy of Standard-of-Care Chemotherapy Combinations in Ewing Sarcoma Cells
Source: Cancers (Basel). 2024 Oct 23;16(21):3565. doi: 10.3390/cancers16213565 (PMC11545275; doi:10.3390/cancers16213565)

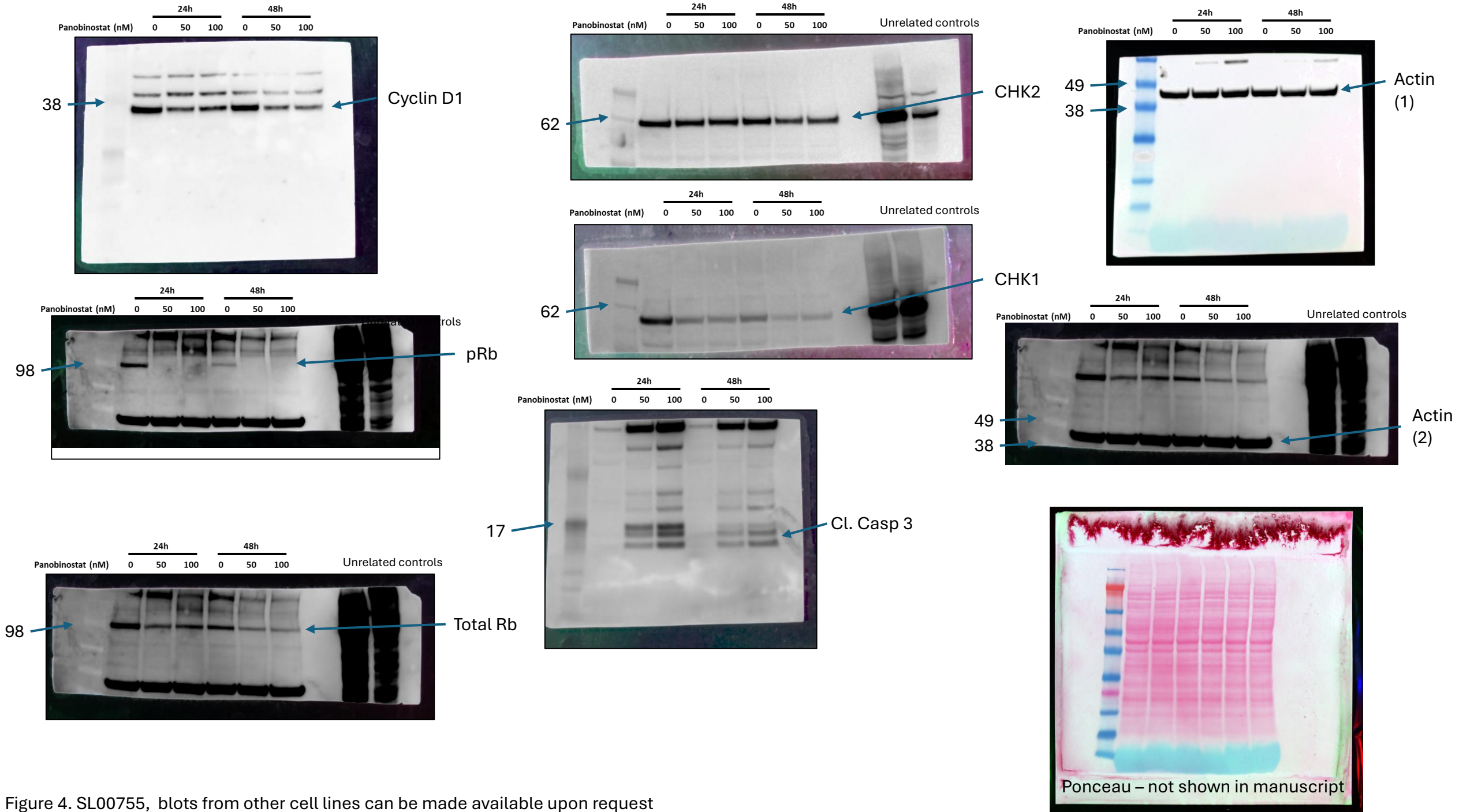

Figure 4. SL00755, blots from other cell lines can be made available upon request

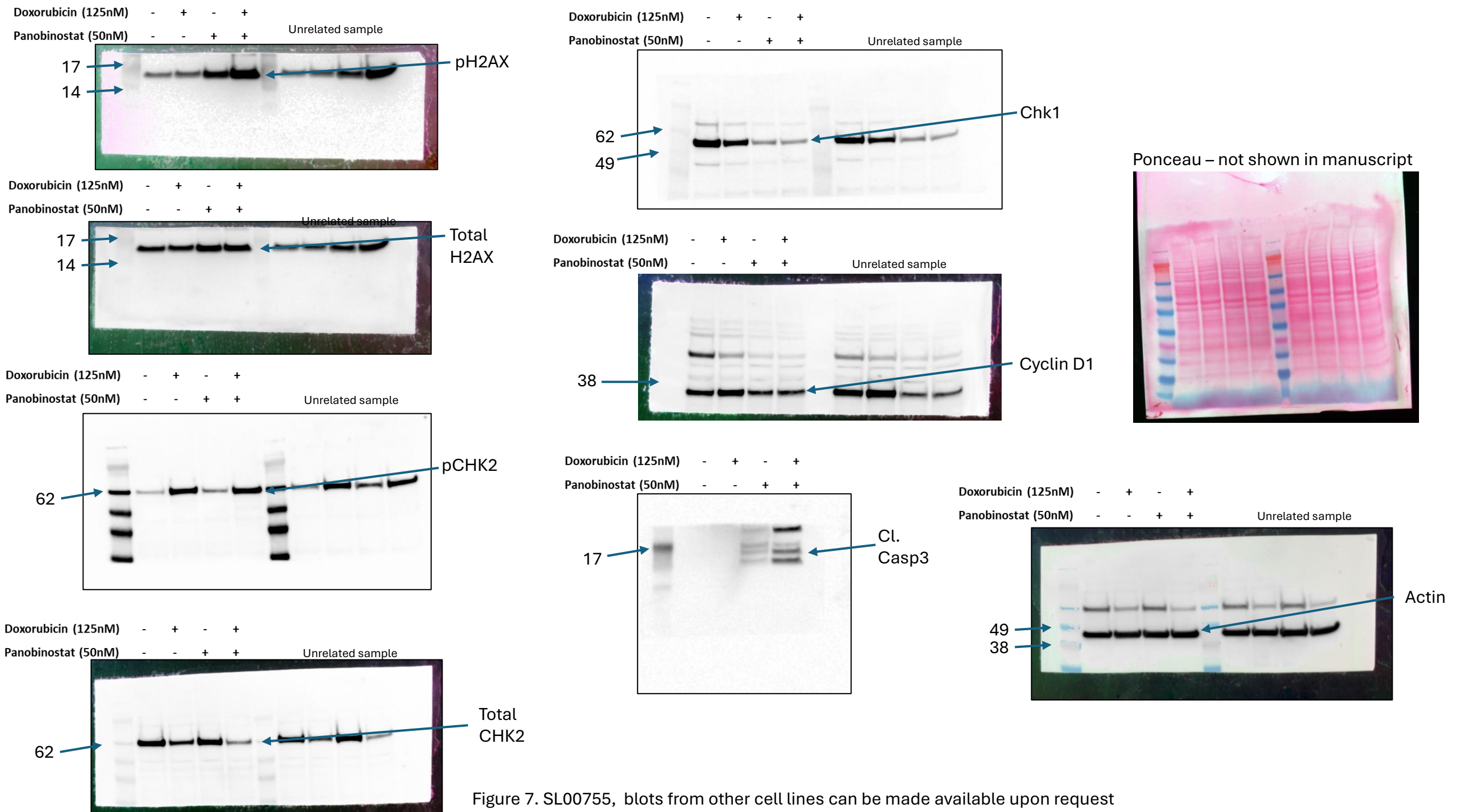

Supplement: Supplementary file 1 [file cancers-16-03565-s001.zip › Figure S4. original-images.pdf]
